# Supplementary material for: Short-Term Effects of Low-Level Ambient Air NO2 on the Risk of Incident Stroke in Enshi City, China
Source: Int J Environ Res Public Health. 2022 May 30;19(11):6683. doi: 10.3390/ijerph19116683 (PMC9180296; doi:10.3390/ijerph19116683)
Supplement: Supplementary file 1 [file ijerph-19-06683-s001.zip › ijerph-1700994-supplementary.pdf]

Table S1. Comparison of ambient NO_2 concentrations in different research areas.

Reference	Study Period	Study Location	Concentration [1]	Descriptive Statistics	Exposure Duration	Type of stroke	Outcome
Amini et al. [1]	1993–1999	Danish	12.6 μg/m³	Mean	Long-term	Ischemic, hemorrhagic	Incidence
Byrne et al. [2]	2013–2017	Dublin, Ireland	20.60 μg/m³	Mean	Short-Term	All types of stroke	Hospital admission
Carlsen et al. [3]	2003–2009	Reykjavik, Iceland	22.10 μg/m³	Mean	Short-Term	All types of stroke	Hospital admission
Yu et al. [4]	1991–1997	Seoul, Korea	22.3 μg/m³	Mean	Short-Term	Ischemic	Mortality
Atkinson et al. [5]	2003–2007	England	22.50 μg/m³	Mean	Long-term	All types of stroke	Incidence
Ho et al. [6]	2009–2018	Singapore	23.90 μg/m³	Mean	Short-Term	Hemorrhagic	Incidence
Byrne et al. [2]	2013–2017	Cork, Ireland	24.00 μg/m³	Mean	Short-Term	All types of stroke	Hospital admission
Kettunen et al. [7]	1998–2004	Helsinki, Finland	27.60μg/m³	Median	Short-Term	All types of stroke	Mortality
Johnson et al. [8]	2007–2009	Canada	15.4 ppb [2]	Mean	medium-term	All types of stroke	Hospital admission
Yorifuji et al. [9]	1999–2009	Shizuoka, Japan	35.11 μg/m³	Mean	Long-term	Ischemic, hemorrhagic	Mortality
Dong et al. [10]	2015–2016	Changzhou, China	38.70 μg/m³	Mean	Short-Term	Ischemic	Incidence, Mortality
Song ea al. [11]	2002–2013	Korea	43.30 μg/m³	Mean	Short-Term	Ischemic	Hospital admission
Guo et al. [12]	2013–2015	Guangzhou, China	44.00 μg/m³	Median	Short-Term	Ischemic	Hospital admission
Qi et al. [13]	2018–2019	Tianjin, China	44.8 μg/m³	Mean	Short-Term	Ischemic	Hospital admission
Villeneuve et al. [14]	1992–2002	Edmonton, Canada	24.00 ppb [3]	Mean	Long-term	All types of stroke	Hospital admission
Crichton et al. [15]	2005–2012	South London, England	45.70 μg/m³	Mean	long-term	Ischemic, hemorrhagic	Incidence
Wang et al. [16]	2008–2014	Shenzhen, China	45.89 μg/m³	Mean	Short-Term	Ischemic	Incidence
Luo et al. [17]	2016–2017	Chengdu, China	47.13 μg/m³	Mean	Short-Term	All types of stroke	Mortality
Huang et al. [18]	2013–2014	Beijing, China	52.50 μg/m³	Mean	Short-Term	All types of stroke	Hospitalization
Xiang et al. [19]	2006–2008	Wuhan, China	53 μg/m³	Mean	Short-Term	All types of stroke	Hospital admission
Tsai et al. [20]	1997–2000	Kaohsiung, Taiwan	28.67 ppb [4]	Median	Short-Term	All types of stroke	Hospital admission

Chen et al. [21]	2013–2015	Jinan, China	57 μg/m³	Mean	Short-Term	All types of stroke	Hospital admission
Hossein et al. [22]	2012–2013	Teheran, Iran	59.0 μg/m³	Mean	Long-term	Ischemic	Hospital admission
Hong et al. [23]	1991–1997	Seoul, Korea	32.20 ppb [5]	Mean	Short-Term	Ischemic, hemorrhagic	Mortality
Lipsett et al. [24]	1996–2005	California, USA	33.59 ppb [6]	Mean	Long-term	All types of stroke	Incidence
Yorifuji et al. [25]	2003–2008	Tokyo, Japan	34.30 ppb [7]	Mean	Short-Term	Hemorrhagic stroke	Mortality

[1] The concentration of air pollution NO_2 during the study period. [2] 15.4 ppb NO_2 is about 28.98 μg/m³. [3] 24.00 ppb NO_2 is about 45.16 μg/m³. [4] 28.67 ppb NO_2 is about 53.95 μg/m³. [5] 32.20 ppb NO_2 is about 60.59 μg/m³. [6] 33.59 ppb NO_2 is about 63.21 μg/m³. [7] 32.20 ppb NO_2 is about 62.48 μg/m³.

Table S2. Descriptive statistics for daily meteorological factors of cold and warm season in Enshi city, 2015–2018.

Meteorological Factors	Season	Mean ± SD	Min	P_{25}	Median	P_{75}	Max
Temperature (°C)	Warm season [1]	20.10 ± 3.92	5.69	17.33	20.56	23.03	27.40
	Cold season [2]	7.65 ± 4.89	−4.02	4.20	6.99	11.46	21.19
Humidity (%)	Warm season	78.38 ± 9.61	42.71	71.58	79.11	86.53	93.75
	Cold season	78.86 ± 9.35	48.20	72.22	80.19	86.91	94.39

[1] Warm season: April, May, June, July, August, and September. [2] Cold season: October, November, January, December, January, February, and March.

Table S3. The demographic information for incidence of stroke in Enshi city, 2015–2018

Classification	Incident Stroke Numbers (%)
Total	9122（100）
Stroke subtypes	
Ischemic	6277（68.81）
Hemorrhagic	2435（26.69）
Other types [1]	410（4.50）
Ages	
Elderly [2]	6111（66.99）
Non-elderly [3]	3011（33.01）
Sexes	
Male	5425（59.47）
Female	3697（40.53）
Seasons	
Warm season [4]	4432（48.59）
Cold season [5]	4690（51.41）

[1] Other types were strokes that do not specifically refer to hemorrhage or ischemia. [2] The age ≥ 65 years were defined as elderly group. [3] The age < 65 years were defined as non-elderly. [4] Warm season: April, May, June, July, August, and September. [5] Cold season: October, November, January, December, January, February, and March.

Table S4. The annual average levels of ambient NO_2 and incident stroke cases in Enshi city during 2015–2018.

Variables	2015	2016	2017	2018	2015–2018
Annual average（μg/m³）	18.96	19.63	23.41	23.61	21.40
Annual incident stroke cases	2683	2433	1448	2558	9122

References

1. Amini, H.; Dehlendorff, C.; Lim, Y. H.; Mehta, A.; Jørgensen, J. T.; Mortensen, L. H.; Westendorp, R.; Hoffmann, B.; Loft, S.; Cole-Hunter, T.; Bräuner, E. V.; Ketzel, M.; Hertel, O.; Brandt, J.; Solvang Jensen, S.; Christensen, J. H.; Geels, C.; Frohn, L. M.; Backalarz, C.; Simonsen, M. K.; Andersen, Z. J., Long-term exposure to air pollution and stroke incidence: A Danish Nurse cohort study. *Environ Int* **2020,** 142, 105891. doi:10.1016/j.envint.2020.105891.
2. Byrne, C. P.; Bennett, K. E.; Hickey, A.; Kavanagh, P.; Broderick, B.; O'Mahony, M.; Williams, D. J., Short-Term Air Pollution as a Risk for Stroke Admission: A Time-Series Analysis. *Cerebrovascular diseases (Basel, Switzerland)* **2020,** 49, (4), 404-411. doi:10.1159/000510080.
3. Carlsen, H. K.; Forsberg, B.; Meister, K.; Gíslason, T.; Oudin, A., Ozone is associated with cardiopulmonary and stroke emergency hospital visits in Reykjavík, Iceland 2003-2009.

Environmental health : a global access science source **2013,** 12, 28. doi:10.1186/1476-069x-12-28.

4. Yu, Y.; Dong, H.; Yao, S.; Ji, M.; Yao, X.; Zhang, Z., Protective Effects of Ambient Ozone on Incidence and Outcomes of Ischemic Stroke in Changzhou, China: A Time-Series Study. *Int J Environ Res Public Health* **2017,** 14, (12). doi:10.3390/ijerph14121610.
5. Atkinson, R. W.; Carey, I. M.; Kent, A. J.; van Staa, T. P.; Anderson, H. R.; Cook, D. G., Long-term exposure to outdoor air pollution and incidence of cardiovascular diseases. *Epidemiology* **2013,** 24, (1), 44-53. doi:10.1097/EDE.0b013e318276ccb8.
6. Ho, A. F. W.; Lim, M. J. R.; Zheng, H.; Leow, A. S.; Tan, B. Y.; Pek, P. P.; Raju, Y.; Seow, W. J.; Yeo, T. T.; Sharma, V. K.; Aik, J.; Ong, M. E. H., Association of ambient air pollution with risk of hemorrhagic stroke: A time-stratified case crossover analysis of the Singapore stroke registry. *International journal of hygiene and environmental health* **2022,** 240, 113908. doi:10.1016/j.ijheh.2021.113908.
7. Kettunen, J.; Lanki, T.; Tiittanen, P.; Aalto, P. P.; Koskentalo, T.; Kulmala, M.; Salomaa, V.; Pekkanen, J., Associations of fine and ultrafine particulate air pollution with stroke mortality in an area of low air pollution levels. *Stroke* **2007,** 38, (3), 918-22. doi:10.1161/01.STR.0000257999.49706.3b.
8. Johnson, J. Y.; Rowe, B. H.; Allen, R. W.; Peters, P. A.; Villeneuve, P. J., A case-control study of medium-term exposure to ambient nitrogen dioxide pollution and hospitalization for stroke. *BMC public health* **2013,** 13, 368. doi:10.1186/1471-2458-13-368.
9. Yorifuji, T.; Kashima, S.; Tsuda, T.; Ishikawa-Takata, K.; Ohta, T.; Tsuruta, K.; Doi, H., Long-term exposure to traffic-related air pollution and the risk of death from hemorrhagic stroke and lung cancer in Shizuoka, Japan. *The Science of the total environment* **2013,** 443, 397-402. doi:10.1016/j.scitotenv.2012.10.088.
10. Dong, H. B.; Yu, Y. Q.; Yao, S.; Lu, Y.; Chen, Z. Y.; Li, G. Y.; Yao, Y.; Yao, X. J.; Wang, S. L.; Zhang, Z., Acute effects of air pollution on ischaemic stroke onset and deaths: a time-series study in Changzhou, China. *BMJ Open* **2018,** 8, (7), 8. doi:10.1136/bmjopen-2017-020425.
11. Song, J.; Lim, Y.; Ko, I.; Kim, J. Y.; Kim, D. K., Association between Air Pollutants and Initial Hospital Admission for Ischemic Stroke in Korea from 2002 to 2013. *Journal of stroke and cerebrovascular diseases : the official journal of National Stroke Association* **2021,** 30, (11), 106080. doi:10.1016/j.jstrokecerebrovasdis.2021.106080.
12. Guo, P.; Wang, Y. L.; Feng, W. R.; Wu, J. G.; Fu, C. X.; Deng, H.; Huang, J.; Wang, L.; Zheng, M. R.; Liu, H. Z., Ambient Air Pollution and Risk for Ischemic Stroke: A Short-Term Exposure Assessment in South China. *Int. J. Environ. Res. Public Health* **2017,** 14, (9), 11. doi:10.3390/ijerph14091091.
13. Qi, X.; Wang, Z.; Guo, X.; Xia, X.; Xue, J.; Jiang, G.; Gu, Y.; Han, S.; Yao, Q.; Cai, Z.; Wang, X.; Wang, L.; Leng, S. X.; Li, X., Short-term effects of outdoor air pollution on acute ischaemic stroke occurrence: a case-crossover study in Tianjin, China. *Occup Environ Med* **2020,** 77, (12), 862-867. doi:10.1136/oemed-2019-106301.
14. Villeneuve, P. J.; Chen, L.; Stieb, D.; Rowe, B. H., Associations between outdoor air pollution and emergency department visits for stroke in Edmonton, Canada. *European journal of epidemiology* **2006,** 21, (9), 689-700. doi:10.1007/s10654-006-9050-9.
15. Crichton, S.; Barratt, B.; Spiridou, A.; Hoang, U.; Liang, S. F.; Kovalchuk, Y.; Beevers, S. D.; Kelly, F. J.; Delaney, B.; Wolfe, C. D., Associations between exhaust and non-exhaust particulate matter and stroke incidence by stroke subtype in South London. *The Science of the*

total environment **2016,** 568, 278-284. doi:10.1016/j.scitotenv.2016.06.009.

16. Wang, Z.; Peng, J.; Liu, P.; Duan, Y.; Huang, S.; Wen, Y.; Liao, Y.; Li, H.; Yan, S.; Cheng, J.; Yin, P., Association between short-term exposure to air pollution and ischemic stroke onset: a time-stratified case-crossover analysis using a distributed lag nonlinear model in Shenzhen, China. *Environmental health : a global access science source* **2020,** 19, (1), 1. doi:10.1186/s12940-019-0557-4.
17. Luo, L.; Dai, Y.; Zhang, F.; Chen, M.; Chen, F.; Qing, F., Time series analysis of ambient air pollution effects on dynamic stroke mortality. *The International journal of health planning and management* **2020,** 35, (1), 79-103. doi:10.1002/hpm.2821.
18. Huang, F.; Luo, Y.; Tan, P.; Xu, Q.; Tao, L.; Guo, J.; Zhang, F.; Xie, X.; Guo, X., Gaseous Air Pollution and the Risk for Stroke Admissions: A Case-Crossover Study in Beijing, China. *Int J Environ Res Public Health* **2017,** 14, (2). doi:10.3390/ijerph14020189.
19. Xiang, H.; Mertz, K. J.; Arena, V. C.; Brink, L. L.; Xu, X.; Bi, Y.; Talbott, E. O., Estimation of short-term effects of air pollution on stroke hospital admissions in Wuhan, China. *PLoS One* **2013,** 8, (4), e61168. doi:10.1371/journal.pone.0061168.
20. Tsai, S. S.; Goggins, W. B.; Chiu, H. F.; Yang, C. Y., Evidence for an association between air pollution and daily stroke admissions in Kaohsiung, Taiwan. *Stroke* **2003,** 34, (11), 2612-6. doi:10.1161/01.Str.0000095564.33543.64.
21. Chen, C.; Wang, X.; Lv, C.; Li, W.; Ma, D.; Zhang, Q.; Dong, L., The effect of air pollution on hospitalization of individuals with respiratory and cardiovascular diseases in Jinan, China. *Medicine (Baltimore)* **2019,** 98, (22), e15634. doi:10.1097/MD.0000000000015634.
22. Alimohammadi, H.; Fakhri, S.; Derakhshanfar, H.; Hosseini-Zijoud, S. M.; Safari, S.; Hatamabadi, H. R., The Effects of Air Pollution on Ischemic Stroke Admission Rate. *Chonnam Med J* **2016,** 52, (1), 53-8. doi:10.4068/cmj.2016.52.1.53.
23. Hong, Y. C.; Lee, J. T.; Kim, H.; Kwon, H. J., Air pollution - A new risk factor in ischemic stroke mortality. *Stroke* **2002,** 33, (9), 2165-2169. doi:10.1161/01.Str.0000026865.52610.5b.
24. Lipsett, M. J.; Ostro, B. D.; Reynolds, P.; Goldberg, D.; Hertz, A.; Jerrett, M.; Smith, D. F.; Garcia, C.; Chang, E. T.; Bernstein, L., Long-term exposure to air pollution and cardiorespiratory disease in the California teachers study cohort. *Am J Respir Crit Care Med* **2011,** 184, (7), 828-35. doi:10.1164/rccm.201012-2082OC.
25. Yorifuji, T.; Kawachi, I.; Sakamoto, T.; Doi, H., Associations of outdoor air pollution with hemorrhagic stroke mortality. *Journal of occupational and environmental medicine* **2011,** 53, (2), 124-6. doi:10.1097/JOM.0b013e3182099175.
